# *Melothria domingensis* (Cucurbitaceae), an endangered Caribbean endemic, is a *Cayaponia*

**DOI:** 10.3897/phytokeys.18.3914

**Published:** 2012-12-06

**Authors:** Hanno Schaefer, Michael H. Nee

**Affiliations:** 1 Plant Biodiversity Research, Technische Universität München, 85354 Freising, Germany; 2 Institute of Systematic Botany, The New York Botanical Garden, Bronx, New York 10458, USA

**Keywords:** Cogniaux, Flora of Hispaniola, *Melothria domingensis*, pollinator shifts

## Abstract

The Neotropical genus *Melothria* (Benincaseae, Cucurbitaceae) is a small group of yellow- or white-flowered climbers with small to medium-sized fruits. In 1899, Alfred Cogniaux described a species from montane rainforest in Haiti as *Melothria domingensis*, presumably based on the overall similarity in habit, leaf shape, and fruit morphology of his incomplete herbarium material to other Central American *Melothria* species. *Melothria domingensis* is still rare in European and American herbaria and the species has never been studied in detail. We here present molecular and morphological analyses, which show that the species is misplaced in *Melothria* and instead belongs in the distantly related tribe Cucurbiteae in the genus *Cayaponia*. We illustrate the species, provide the formal transfer and an extended description, and discuss the phylogenetic, biogeographic and ecological implications, including the finding that most likely bee- and not bat-pollination is ancestral in *Cayaponia*.

## Introduction

The genus *Melothria* L. (including *Melancium* Naudin, *Cucumeropsis* Naudin, and *Posadaea* Cogn.) includes 12–15 species, confined to arid plains, clearings and forest margins, grass- or woodlands from the southern United States through Central and South America down to northern Argentina (Schaefer and Renner 2011 a, b). One species, *Melothria sphaerocarpa* (Cogn.) H.Schaef. & S.S.Renner, is also found in tropical West Africa, where it might have arrived only recently through human-mediated transport (Schaefer and Renner 2010); and another species, *Melothria pendula* L., is locally invasive in tropical Asia (De Wilde and Duyfjes 2010). *Melothria* species are monoecious, small to medium-sized, herbaceous climbers with usually simple leaves, small yellow or white flowers, and the fruit a smooth, and often fleshy berry to 20 cm in *Melothria sphaerocarpa*. Each fruit contains numerous, strongly compressed seeds; and the testa tends to be smooth, white, and often covered by long appressed hairs (Schaefer and Renner 2011b).

In 1899, Alfred Cogniaux described a new species of *Melothria* based on herbarium material from the Caribbean island of Hispaniola (Haiti and the Dominican Republic), which he had received from the Krug and Urban collection (Urban 1899). The type material (*L. Picarda 1503*, BR) contains complete leaves and fruit plus seeds but no fully developed flowers. While the overall habit, leaf shape and fruit type are quite similar to Central American species of *Melothria*, the ovoid seeds would be unique in that genus and are very similar to another, very distantly related genus: *Cayaponia* Silva Manso in the tribe Cucurbiteae. And indeed, the Brazilian *Cayaponia* expert Vera Gomes-Klein annotated a specimen of *Melothria domingensis* Cogn. in the Berlin Herbarium (*E. L. Ekman 1319*, B) as “*Cayaponia* sp.” in July 1992; and two collections at New York Botanical Garden (*P. Acevedo-Rodríguez et al. 13011 and 13274*, NY) had originally been identified as *Cayaponia racemosa* (Mill.) Cogn. by M. T. Strong in 2010. The aim of our study here is to test this hypothesis using molecular and morphological data and to find out whether *Melothria domingensis* is indeed a misplaced *Cayaponia* or alternatively represents a unique type of seed morphology in *Melothria*.

## Materials and method

**Molecular analyses.**Total genomic DNA was isolated from leaf samples of four herbarium specimens of *Melothria domingensis* (all collected in the Dominican Republic, Table 1) using a commercial plant DNA extraction kit (NucleoSpin, MACHEREY-NAGEL, Düren, Germany), and following the manufacturer’s manual. Polymerase chain reactions (PCR) following standard procedures were used to amplify the entire nuclear ribosomal ITS region plus the following five chloroplast regions: *rbcL* gene, *trnL* intron, and the three intergenic spacer regions *trnL-trnF*, *rpl20-rps12*, and *trnH-psbA*. PCR protocols and primers are given in Kocyan et al. (2007) and Schaefer et al. (2009). In addition, we used the primer pair *trnH* (5’- CGC GCA TGG TGG ATT CAC AAA TC) and *psbA* (5’- GTT ATG CAT GAA CGT AAT GCT C) designed by Sang et al. (1997) with an annealing temperature of 48°C to amplify the *trnH-psbA* spacer region. Crude PCR products were sent to Functional Biosciences, Inc. (Madison, WI, USA) for ExoSap cleaning and Sanger sequencing with the same primers used for PCR reactions. Seventeen sequences were newly generated for *Melothria domingensis* plus eleven ITS sequences for selected species of the genera *Abobra* Naudin, *Calycophysum* H. Karst & Triana, *Cionosicys* Griseb., *Schizocarpum* Schrad., and *Tecunumania* Standl. & Steyerm., all of the tribe Cucurbiteae, to obtain a more balanced sampling in our ITS matrix (for GenBank accession numbers and vouchers see table 1). Raw sequences were edited with Sequencher 4.9 (Gene Codes, Ann Arbor, Michigan, USA) and aligned by eye, using MacClade 4.08 (Maddison and Maddison 2003). We then added to those alignments sequences for all *Cayaponia* species available on Genbank (mainly from Kocyan et al. (2007), Schaefer et al. (2009), and Duchén and Renner (2010)) plus a set of Cucurbitaceae species representing all genera of the tribe Cucurbiteae, three representatives of *Melothria* and representatives of the genera containing Caribbean species based on Schaefer et al. (2008). Bayesian and maximum likelihood analyses were performed with a final dataset of 67 accessions representing 60 species.Data matrix and trees have been deposited in TreeBASE (http://www.treebase.org/ ) study number S13322.

**Table 1.** Taxa, Genbank accession numbers, and voucher information.

Taxon	Geographic origin	Specimen voucher	rbcL	trnL	trnL-trnF	rpl20-rps12	trnH-psbA	ITS1-5.8S-ITS2
*Abobra tenuifolia*	Argentina, Entre Rios	T.M.Pedersen 10287 (GH)	--	--	--	--	--	JX505456
*Calycophysum weberbaueri*	Peru, Cuzco	collector unknown (GH)	--	--	--	--	--	JX505457
*Cayaponia domingensis*	Hispaniola, Dominican Republic	G.J.Gastony et al. 640 (GH)	--	JX505449	JX505445	JX505453	JX505473	JX505465
*Cayaponia domingensis*	Hispaniola, Dominican Republic	P.Acevedo-Rodríguez et al. 13274 (GH)	JX505455	JX505451	JX505447	JX505452	JX505471	JX505464
*Cayaponia domingensis*	Hispaniola, Dominican Republic	A.H.Liogier 12563 (GH)	--	--	--	JX505454	JX505472	JX505466
*Cayaponia domingensis*	Hispaniola, Dominican Republic	A.H.Liogier 12908 (GH)	--	JX505450	JX505446	--	--	JX505467
*Cayaponia (Selysia) prunifera*	Suriname	Lindemann et al. 398 (NY)	--	--	--	--	--	JX505458
*Cionosicys excisus*	Mexico	E.Cabrera 15257 (GH)	--	JX505448	JX505444	--	--	JX505459
*Cionosicys excisus*	Mexico	G.F.Gauner 888 (GH)	--	--	--	--	--	JX505460
*Cionosicys macranthus*	Honduras	A.Molina 3838 (GH)	--	--	--	--	--	JX505461
*Cionosicys macranthus*	Mexico	D.M.Kearns 321 (GH)	--	--	--	--	--	JX505462
*Cionosicys pomiformis*	Jamaica	R.A.Howard & G.R.Proctor 14941 (GH)	--	--	--	--	--	JX505463
*Tecunumania quetzalteca*	Costa Rica	R.W.Lent 2311 (GH)	--	--	--	--	--	JX505470
*Schizocarpum palmeri*	Mexico, Rio Mayo	H.S.Gentry 1032 (GH)	--	--	--	--	--	JX505468
*Schizocarpum reflexum*	Mexico, Michoacán	M.Porter 1377 (GH)	--	--	--	--	--	JX505469

Maximum likelihood (ML) analyses and non-parametric bootstrap searches (BS) with the fast-bootstrap algorithm were performed using RAxML-VI-HPC v. XX (Stamatakis et al. 2008). RAxML searches relied on the GTR + G + I model and model parameters were estimated over the duration of specified runs. Bayesian inference also used the GTR + G model (with the default four rate categories) and relied on MrBayes v. 3.2.1 x64 (Ronquist and Huelsenbeck 2003). We analyzed the combined dataset with two partitions (plastid and nuclear ITS), allowing partition models to vary by unlinking gamma shapes, transition matrices, and proportions of invariable sites. Markov chain Monte Carlo (MCMC) runs started from independent random trees, were repeated twice, and extended for ten million generations, with trees sampled every 1000th generation. Convergence was assessed using Tracer 1.5 (Rambaut and Drummond 2003). Trees saved prior to convergence were discarded as burn-in (2000 trees), and a consensus tree was built from the remaining trees.

**Morphological analyses.** We studied *Melothria domingensis* specimens from the following herbaria: BR, GH, NY, U, and US. For comparison, both authors also studied a large number of *Cayaponia* and other Neotropical Cucurbitaceae specimens from all major European and American herbaria over the past decade. All measurements given in the text are from dry herbarium specimens.

**Analysis of pollination syndrome evolution and biogeography.**We used the same approach as described in Duchén and Renner (2010), namely ancestral character state reconstruction under maximum likelihood in Mesquite v. 2.72 (Maddison and Maddison 2009) based on the Markov k-state one-parameter model. We added *Melothria domingensis* to the matrix of that previous study with the character states “bee pollination” (based on flower morphology) and “rainforest habitat” (based on herbarium label information).

## Results

### Molecular analyses

Analyses of the nuclear ITS data matrix (55 accessions, 984 aligned nucleotides) and the individual and combined plastid matrices (67 accessions, 4831 aligned nucleotides) produced congruent phylogeny estimates (Fig. 2, Fig. 3; phylogenies for individual plastid marker not shown), with all areas of discordance being restricted to branches with low support (i.e., BS <60% and Bayesian posterior probability (PP) <0.9). We therefore combined the ITS and plastid matrices into a single matrix (68 accessions, 60 species, 5673 aligned nucleotides) and in the following focus in our discussion on the phylogeny estimate built using this largest matrix (Fig. 4): the different accessions of *Melothria domingensis* are almost identical in their sequences and group together with high support (100% BS, 1.0 PP, Fig. 4). They are deeply nested in *Cayaponia*, which is monophyletic after inclusion of *Selysia* Cogn. as already suggested by Duchén and Renner 2010 (100% BS, 1.0 PP, Fig. 4). Sister group to *Cayaponia* is the monotypic *Abobra* (79% BS, 1.0 PP, Fig. 4) and the two together are sister to the *Schizocarpum* clade (100% BS, but <0.9 PP, Fig. 4). Within *Cayaponia*, *Melothria domingensis* is placed in a grade with the North American *Cayaponia quinqueloba* (Raf.) Shinners (syn. *Cayaponia grandifolia* Torrey & A. Gray) and a large unresolved group including *Cayaponia podantha* Cogn., *Cayaponia americana* Cogn., *Cayaponia africana* (Hook.f.) Exell and several other Central and South American species. In contrast the three other *Melothria* species included in our matrix form a highly supported clade (100% BS, 1.0 PP, Fig. 4) and are sister to *Cucumis melo* L. (98% BS, 1.0 PP, Fig. 4).

### Morphological comparison

Comparison of seed, floral, fruit, and vegetative characters of *Melothria domingensis* with all available *Cayaponia*, *Cionosicys*, and *Melothria* material reveals that seed number, size and shape are most similar to *Cayaponia* and not to *Melothria* or *Cionosicys*. Mottled fruits like those of *Melothria domingensis* also occur in *Cionosicys*, but all currently known species of *Cionosicys* have much larger fruits and are many-seeded. In *Melothria*, fruits can be small and striped or mottled but all currently known species of that genus have many-seeded fruits with strongly compressed seeds. Only in *Cayaponia* do we find small fruits with few, tumid seeds.

Within *Cayaponia*, the relatively small leaves and few-flowered fascicles of *Melothria domingensis* are most similar to *Cayaponia quinqueloba*, an endemic from the southeastern United States. The fruits of *Cayaponia quinqueloba*, however, have the typical coriaceous or chartaceous exocarp of many other *Cayaponia* species, and not the distinctive spots of *Melothria domingensis* fruits. Another *Cayaponia* species with mottled fruits is *Cayaponia tibiricae* (Naudin) Cogn. (syn. *Cayaponia martiana* (Cogn.) Cogn.) of eastern Brazil, but its inflorescence is branched like that of *Cayaponia racemosa* (Mill.) Cogn. and a few others.

### New combination and extended description

Since molecular and morphological analyses agree that *Melothria domingensis* should be placed in the genus *Cayaponia*, we here provide the necessary new combination followed by an extended description:

#### Cayaponia domingensis

(Cogn.) H.Schaef. & M.Nee comb. nov.

urn:lsid:ipni.org:names:77123716-1

http://species-id.net/wiki/Cayaponia_domingensis

Melothria domingensis Cogn. in Urb., Symb. Ant. 1: 451. 1899. TYPE: Haïti: Ouest: prope Port-au-Prince in montibus Furcy del Tête, bois de pin [west Haiti, near Port-au-Prince, Furcy del Tête mountain, pine forest], 1800 m. Nov 1896, *L. Picarda 1503*, BR. [basionym]

##### Description.

**Figure 1.**
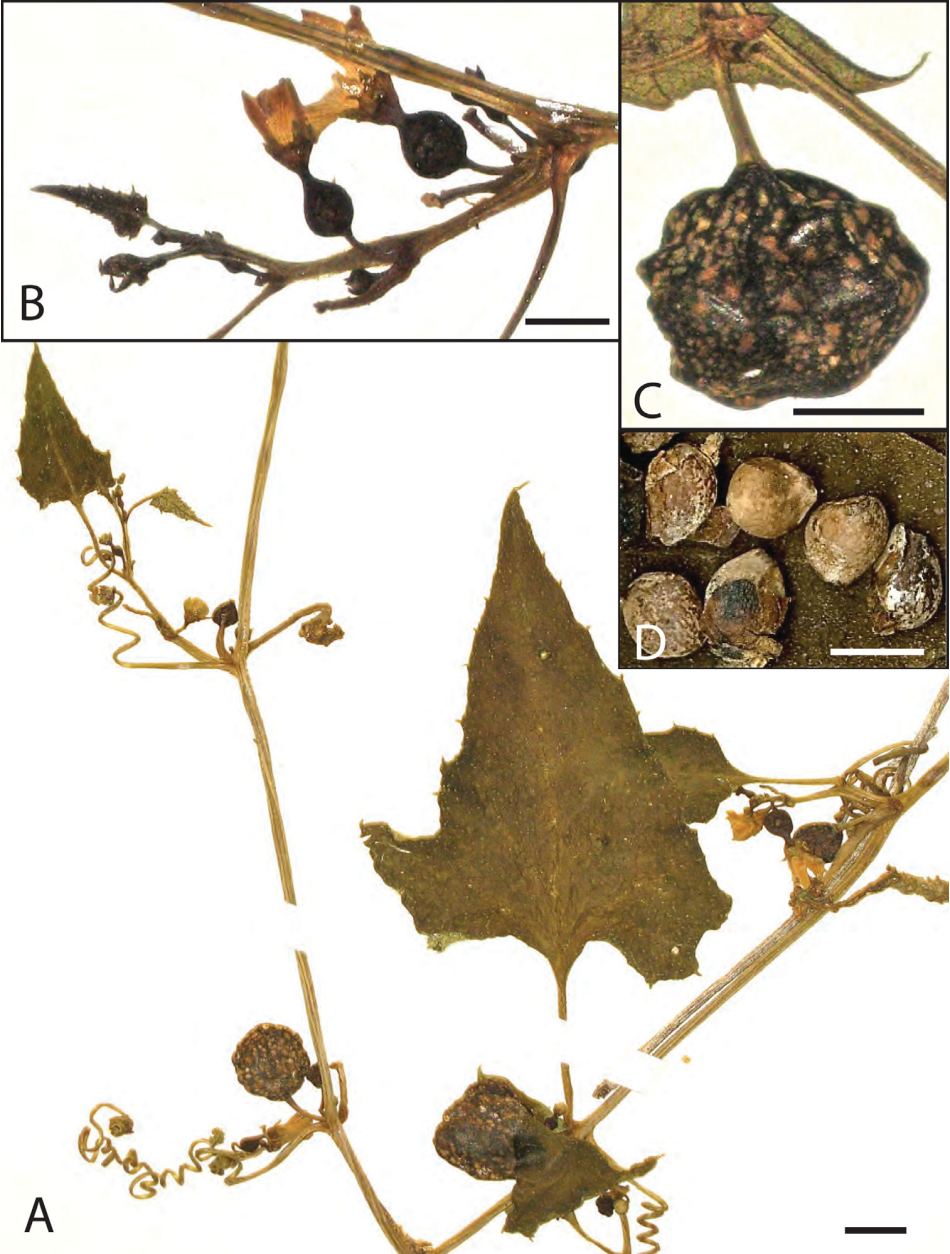
*Cayaponia domingensis* (Cogn.) H. Schaef. & M. Nee **A** habitus with leaves, tendrils, staminate and pistillate flowers, and young fruits **B** pistillate flowers **C** fruit **D** seeds; scale bar: 5 mm. Photographs of A.H.Liogier 12563 (GH) by H. Schaefer.

Monoecious climber with 3–4 m long, slender, grooved stems (Fig. 1A); leaves triangulate-cordate, often deeply 3(-5)-lobed, shortly tomentose on both sides, glabrescent above, margin finely dentate; petioles 10–20 mm, striate; tendrils simple. Flowers probably diurnal (based on herbarium specimens with open flowers), solitary or in few-flowered fascicles in the leaf axils (Fig. 1B), the staminate and pistillate coaxillary, shortly pedicillate; corolla pale yellow, inner petal surface white; calyx glabrous, broadly campanulate; stamens inserted near the base of the tube; filaments distinct, two anthers 2-thecous, one 1-thecous; ovary globose, glabrous; style erect, linear, inserted on a basal nectary; stigmas 3, dilated, reflexed; staminodes 3, minute; fruit globose, glabrous, 10–20 mm diam., with thin, leathery wall, dark green turning orange with greenish white mottling; fruiting pedicels 10–30 mm long (Fig. 1C); seeds 6, broadly ovoid, 5 × 4 × 2.5 mm; testa light brown, smooth (Fig. 1D).

##### Phenology.

Flowering and fruiting specimens have been collected in April, June, August and September.

##### Distribution.

Endemic to the Greater Antilles, island of Hispaniola, and perhaps Puerto Rico (one record from Ponce, Toro Negro Commonwealth Forest, between Cerro Maravillas and Monte Jayuya, along road off highway 143, 1190–1200 m, 26 Feb 1993, *Breckon et al. 4427*, NY, specimen not seen).

##### Habitat.

According to specimen label information, the species occurs in rainforest, thickets and disturbed areas of cloud forest 1200–2200 m a.s.l., on limestone rocks with *Ardisia* Gaertn., *Brunellia* Ruiz & Pav., *Buddleja* L., *Cestrum* L., *Coccoloba* P.Browne, *Cordia* L., *Daphnopsis* Mart., *Garrya* Douglas ex Lindl., *Heterotrichum* M.Bieb., *Lobelia* L., *Meliosma* Blume, *Miconia* Ruiz & Pav., *Myrsine* L., *Palicourea* Aubl., *Persea* Mill., *Ocotea* Aubl., *Trema* Lour., *Turpinia* Raf., and *Weinmannia* L.

##### Conservation status.

Listed as in danger of extinction (“peligro de extinción”) on an informal webpage for the flora and fauna of Hispaniola (Marcano 2009). The available information on distribution, population size, and threats does not seem sufficient for a formal classification following IUCN red list criteria.

##### Local names.

Mirliton blanc, Mirliton marron, Mirliton sauvage (Barker and Dardeau 1930; Liogier 1986).

##### Specimens examined.

Dominican Republic. Baoruco: Sierra de Neiba, Sabana del Silencio, 18°39'07"N, 71°33'26"W, 2201 m, 19 Jun 2003 (fl., fr.), P.Acevedo-Rodríguez et al. 13011 (NY); Elías Peña:  Sierra de Neiba, near La Doscientos, Hondo Valle, 1750–1850 m, 5–7 Sep 1968, A.Liogier 12501 (NY, US); Sierra de Neiba, near La Doscientos, S of Hondo Valle, 1750–1850 m, 5–7 Sep 1968, A.Liogier 12563 (GH, NY). Independencia: Sierra de Neiba, between Ángel Félix and Aniceto Martínez, 18°41'37"N, 71°46'56"W, 1867 m, 24 Jun 2003 (fl., fr.), P.Acevedo-Rodríguez et al. 13274 (GH, NY); Sierra de Neiba, along the Carretera Internacional near the crest of the range, along the Haitian border, vic. the San Rafael and Independencia border, 18°39'00"N, 071°37'48"W, 1700–2000 m, 9 Aug 1967, G.J.Gastony, G.C.Jones & D.H.Norris 640 (GH, NY, US); near Sapotén, above El Aquacate, Duvergé, 1500–1700 m, 4–5 Jan 1972, A.Liogier 18359 (F, NY); Sapotén, El Aguacate, Duvergé, 1300 m, 25 Jun 1977, A.Liogier & Liogier 27009 (NY); Sierra de Neiba, 30 km above Sabana Real on road paralleling the Haïtian border, 18°40'00"N, 71°46'00"W, 1845 m, 18 Apr 2003 (fl., fr.), M.Nee & J.C.Montero Castro 52304 (MO, NY). La Vega: near La Ciénaga, N of Constanza, 1700 m, 16 May 1959, Jiménez 4010 (US); La Nevera, from Valle Nuevo to San José de Ocoa, 2100 m, 18 Oct 1968, A.Liogier 13134 (NY); cabezadas de Ciénaga de la Culata, Constanza, 1650 m, 16 Oct 1968, A.Liogier 13072 (NY); Loma Redonda, Ciénaga de la Culata, Constanza, 1600–1950 m, 23 Sept 1969, A.Liogier 16015 (NY, US). Pederales: Sierra de Bahoruco, sección Los Arroyos, 18°25'54"N, 71°74'45"W, 1500 m, 10 Jul 2007 (fl., fr.), T.Clase, L.Raz, D.Castillo, L.Reinoso & E.Soto. 4539 (MO); above Los Arroyos, along the International Highway from Pedernales to Duvergé, 1500–1600 m, 8 Nov 1969, A.Liogier 16775 (NY, US). Peravia [now San José de Ocoa]: 33.9 mi. N of the Parque Central de San José de Ocoa on the road to Constanza, 1800 m, 7 Jul 1982, T.A.Zanoni, M.M.Mejía & J.D.Pimentel B. 21376 (MO, NY, U). San José de Ocoa: La Horma Arriba, 1800–2000 m, 1 May 1972, A.Liogier 18595 (F, NY); La Nevera, 2100 m, 22–23 Oct 1976, A.Liogier & Liogier 25690 (NY). Santiago: upper Río Bao valley, at the base of La Pelona, 1700 m, 1–7, A.Liogier 12908 (NY). Haïti. Ouest: Fourcey, Massif de la Selle, ca. 1750 m, 5 Aug. 1924 (fl., fr.), E.L.Ekman 1319 (B, S, US). Massif de la Selle; Parc National Morne la Visite; Morne d›Enfer, eastern slopes and ridge, 1850–1880 m, 14 May 1984, W.S.Judd 4669 (FLAS).

**Figure 2.**
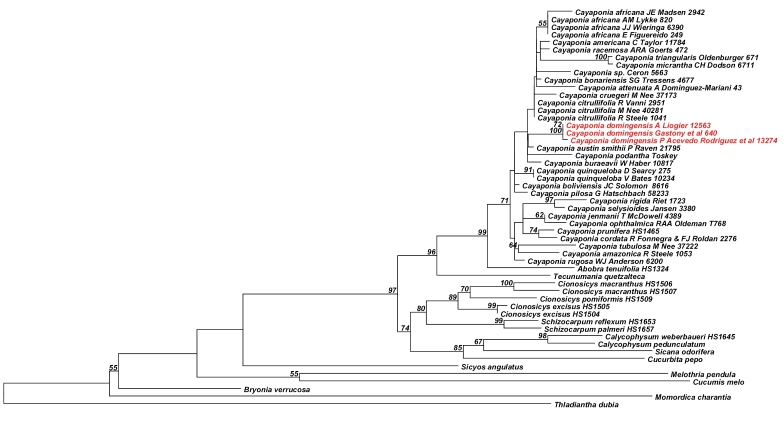
Best Maximum likelihood tree for *Cayaponia* and relatives based on the nuclear ribosomal ITS region (55 accessions, 984 aligned nucleotides). Likelihood bootstrap values ~ 60% is indicated above branches.

**Figure 3.**
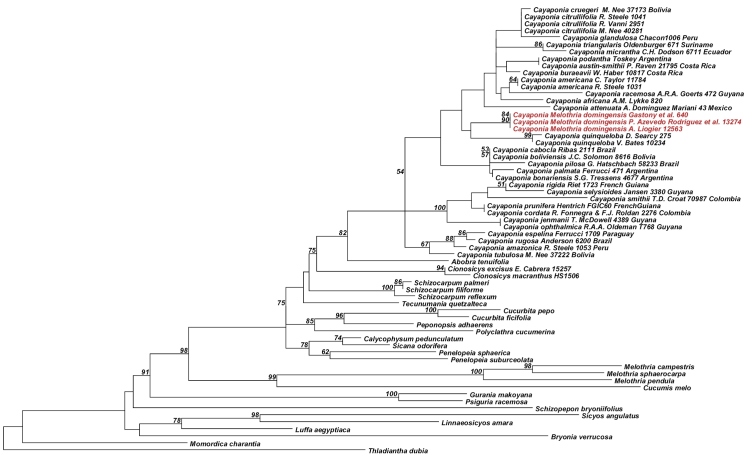
Best Maximum likelihood tree for *Cayaponia* and relatives based on six combined chloroplast loci: *rbcL* gene, *trnL* intron, *trnL-F* spacer, *rpl20-rps12* spacer, and *trnH-psbA* spacer (64 accessions, 4831 aligned nucleotides). Likelihood bootstrap support ~ 60% is indicated above branches.

**Figure 4.**
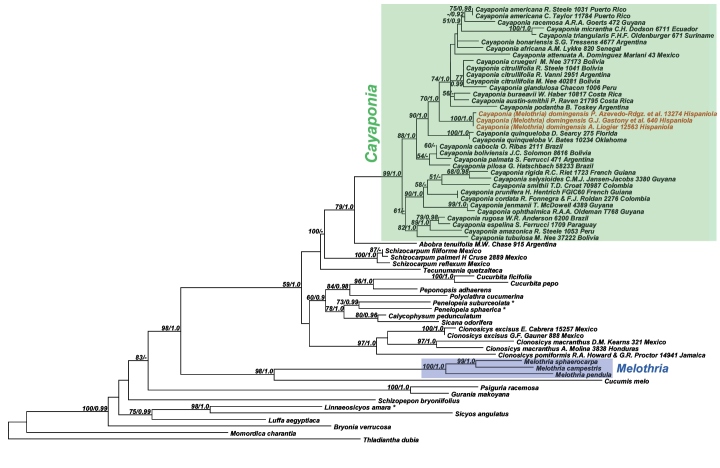
Best Maximum likelihood tree for *Cayaponia* and relatives based on 973 nucleotides from ITS plus combined chloroplast loci (67 accessions, 5673 aligned nucleotides). Likelihood bootstrap support ~ 60% and Bayesian posterior probabilities ~ 0.9 are indicated at the nodes.

### Pollination syndrome evolution

**Figure 5.**
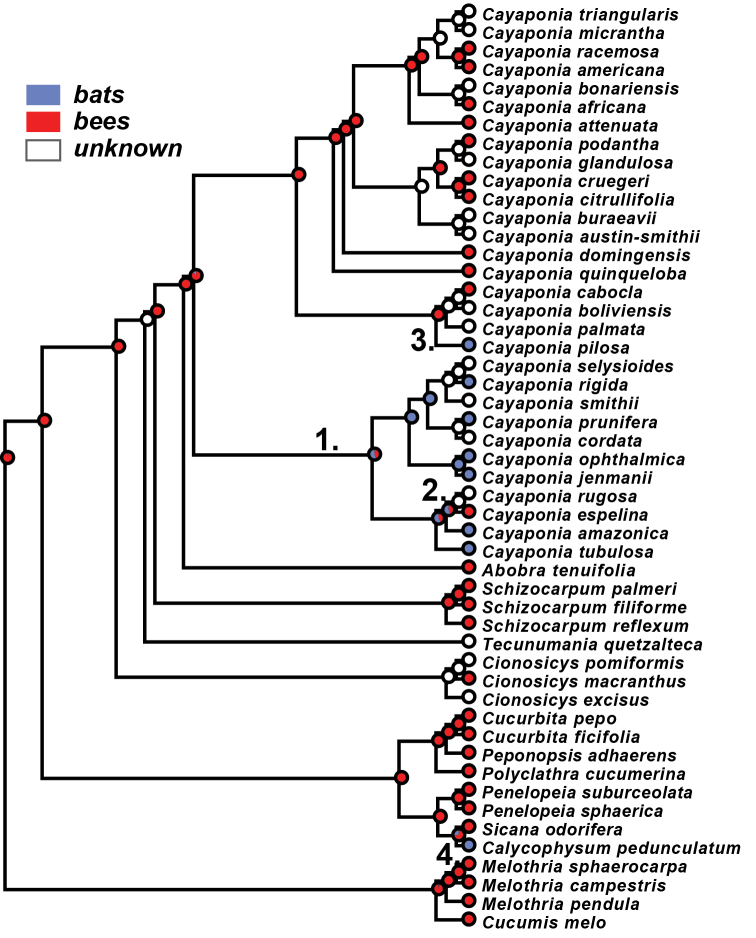
Maximum likelihood reconstruction of ancestral pollination syndromes: bat pollination – blue; bee pollination – red; unknown – white; inferred pollinator shifts numbered 1–4.

Our re-analysis of the evolution of pollination syndromes in *Cayaponia* reveals that bee pollination is most likely the ancestral state for the clade (Fig. 5) followed by one shift to bat pollination along the stem of the *Cayaponia tubulosa* Cogn.*-C. prunifera* (Poepp. & Endl.) P.Duchén & S.S.Renner clade and one reversal to bee pollination within the same clade (*Cayaponia espelina* (Silva Manso) Cogn.). A third shift is inferred for *Cayaponia pilosa* Cogn., which is bat pollinated but nested in a bee pollinated clade (Fig. 5).

### Ancestral habitat reconstruction

**Figure 6.**
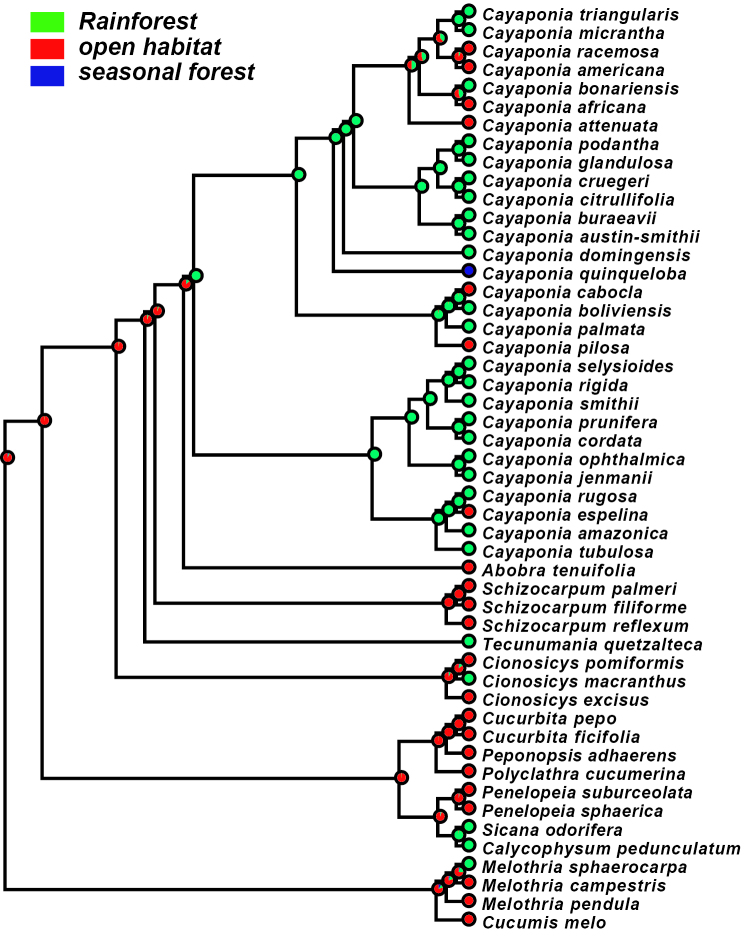
Maximum likelihood reconstruction of ancestral habitat type: rainforest - green; open habitat (including cerrado, savanna, bushland) - red; seasonal forest - blue.

We find that the ancestral habitat of the *Cayaponia* lineage was most likely rainforest, which is in agreement with most extant species still being confined to some type of rainforest habitat (Fig. 6). Only few species, including *Cayaponia espelina*, *Cayaponia pilosa*, and some members of the clade containing *Cayaponia attenuata* (Hook. & Arn.) Cogn. shifted to more open habitats (Fig. 6).

## Discussion

Molecular and morphological evidence both show that *Melothria domingensis* is best placed in the *Cayaponia* clade and probably close to the North American *Cayaponia quinqueloba*. However, due to the poor resolution in parts of our *Cayaponia* phylogeny, we are unable to identify exact sister group relationships. Better taxon sampling and additional variable DNA markers are needed to solve this question.

The newly identified *Cayaponia* species from Hispaniola is a rain- or cloud forest inhabitant with small, probably diurnal flowers on short pedicels that match all characters for bee-pollination in *Cayaponia* discussed in Duchén and Renner (2010). It therefore does not fit to the pattern of pollinator shifts from bat- to bee-pollination as an adaptation to open savanna habitats hypothesized for *Cayaponia* by Duchén and Renner (2010) based on an analysis including 19 of the c. 60 species of *Cayaponia*. And indeed, our re-analysis of the pollination syndrome evolution in *Cayaponia* also questions the finding that bat pollination might be the ancestral condition in the clade. We find that based on the extended sampling, the most likely ancestral condition is bee pollination but with still 70% of the currently known *Cayaponia* species remaining to be sequenced, this pattern can easily change in the near future. We can confirm, however, at least one inferred shift from bat- to bee-pollination in *Cayaponia espelina*, which would be one of very few transitions in that direction (Duchén and Renner 2010; Van der Niet and Johnson 2012). Fieldwork on pollinators of *Cayaponia espelina*, a common species of the Brazilian Cerrado, would be needed to elucidate this interesting case further.

*Cayaponia domingensis* is one of only few species in the genus and in the entire family Cucurbitaceae that occur above 2000 m a.s.l. (the highest collections known are from 2100 m a.s.l., *Liogier & Liogier 25690* (NY), *A. Liogier 13134* (NY) and 2201 m a.s.l., *P. Acevedo-Rodríguez et al. 13011* (NY)). The mechanisms that allow this species to thrive at those altitudes are unknown but might be interesting to study. Most Cucurbitaceae are highly frost-sensitive and survive cold periods only as seeds (e.g. North American *Sicyos angulatus* L.), rootstocks (e.g. Central European *Bryonia dioica* Jacq.), or tubers (e.g. *Thladiantha dubia* Bunge). The only perennial Cucurbitaceae that thrive at even higher altitudes are some Himalayan *Thladiantha* species that reach up to 3500 m a.s.l.

The mottled fruits characteristic for *Cayaponia domingensis* are very rare in the genus and might have evolved as an adaptation to a specialised seed dispersal agent on the island. Unfortunately, we lack field observations on dispersal agents and judging from fruit and seed size, a wide range of animals, including birds, lizards, and small mammals might be involved.

Recent transoceanic dispersal has been inferred for the ancestors of *Cayaponia africana* s.l. (Schaefer et al. 2009; Duchén and Renner 2010). While Duchén and Renner (2010) hypothesized a stepping-stone route via the Brazilian island Fernando de Noronha, our findings suggest that long-distance dispersal out of the Caribbean might be another option.

Our results highlight that even after two decades of Molecular Systematics, we still need more sequencing combined with morphological analyses to sort out taxonomic problems in Cucurbitaceae and other understudied families. We can now be confident about the phylogenetic position of this enigmatic Caribbean endemic. This is, however, only a first step and we now plan to do fieldwork on Hispaniola to obtain accurate distribution data, find out more about the ecology of this species, identify the threats to the remaining populations and ultimately develop a management plant to guarantee the survival of this unique endemic.

## Supplementary Material

XML Treatment for Cayaponia domingensis
